# The impacts and implications of the community face mask use during the Covid‐19 pandemic: A qualitative narrative interview study

**DOI:** 10.1111/hex.13752

**Published:** 2023-03-21

**Authors:** Esmée Hanna, Graham Martin, Anne Campbell, Paris Connolly, Kristine Fearon

**Affiliations:** ^1^ Leicester School of Allied Health Sciences De Montfort University Leicester UK; ^2^ The Healthcare Improvement Studies Institute Cambridge University Cambridge UK; ^3^ NIHR Health Protection Research Unit in Healthcare Associated Infections and Antimicrobial Resistance (NIHR HPRU AMR) in the Faculty of Medicine Imperial College London London UK; ^4^ Centre for Reproduction Research De Montfort University Leicester UK

**Keywords:** Covid‐19, face masks, intervention acceptability, public health, qualitative research

## Abstract

**Introduction:**

A range of nonpharmaceutical public health interventions has been introduced in many countries following the rapid spread of Covid‐19 since 2020, including recommendations or mandates for the use of face masks or coverings in the community. While the effectiveness of face masks in reducing Covid‐19 transmission has been extensively discussed, scant attention has been paid to the lived experience of those wearing face masks.

**Method:**

Drawing on 40 narrative interviews with a purposive sample of people in the United Kingdom, with a particular focus on marginalised and minoritized groups, our paper explores experiences of face mask use during the pandemic.

**Results:**

We find that face masks have a range of societal, health and safety impacts, and prompted positive and negative emotional responses for users. We map our findings onto Lorenc and Oliver's framework for intervention risks. We suggest that qualitative data offer particular insights into the experiences of public health interventions, allowing the potential downsides and risks of interventions to be more fully considered and informing public health policies that might avoid inadvertent harm, particularly towards marginalised groups.

**Patient or Public Contribution:**

The study primarily involved members of the public in the conduct of the research, namely through participation in interviews (email and telephone). The conception for the study involved extensive discussions on social media with a range of people, and we received input and ideas from presentations we delivered on the preliminary analysis.

## 1 BACKGROUND

The use of face masks during the Covid‐19 pandemic has been the subject of much scientific debate and discussion, largely around their effectiveness in breaking the transmission of the severe acute respiratory syndrome coronavirus 2.^1^, ^2^ Masks first became mandatory in England in mid‐June 2020, with wider mandating across the United Kingdom during the summer of 2020, and while mandates have come and gone with successive waves and variants, masks have become a routine part of living with and through a pandemic for many.^3^ The World Health Organisation recommended that masks be used as part of a range of nonpharmaceutical interventions against Covid‐19.^4^ Not everyone will be able to wear a face mask, for various reasons (including physical and mental health conditions and disabilities), although the criteria for and approach to granting exemptions varies across countries and jurisdictions,^5^ in some cases changing through time.^6^

Masks and other forms of face covering already had a longstanding use in some East Asian countries and appeared to be adopted more readily in these settings during the onset of the Covid‐19 pandemic.^7^ However, in the United Kingdom, where the use of masks in daily life is—or was—not common practice, the introduction of face masks generated significant public debate and discussion.^8^ In healthcare settings, face masks have been reported to cause physical issues for users, such as headaches and shortness of breath, which could be problematic for those with existing health conditions.^9^ Some work around gendered perceptions of mask use has also been published, suggesting men are more likely to see masks as compromising their independence, whereas women are more likely to report discomfort.^10^ However, beyond these studies, there has been little attention to experiences of the use of masks in the community and people's perceptions of this public health intervention.

Given this limited evidence base, concern has been raised regarding the impact of face masks on particular groups during the pandemic,^11^ notably those with disabilities who may face social exclusion as a result of mandatory mask policies.^12^ Bakhit et al. identified 37 studies in their systematic review on the possible downsides of mask use, concluding that greater research is urgently needed to explore these downsides and strategies to mitigate against them.^13^ Given the likelihood of future pandemic events,^14^ and the need for ongoing community containment of Covid‐19 face masks are likely to be a feature of the public health toolkit across the world for the foreseeable future, and so understanding the experience of their use is vitally important. Our study examines the experiences of a diverse sample of face mask users, with a particular focus on those who may be at risk of negative impacts, as well as those who are exempt from mask‐wearing, to explore the lived experience of face mask use.

## 2 METHODS

### 2.1 Materials

The aim of the study was ‘to understand the lived experience of face masks during the Covid‐19 pandemic across different groups’ in the United Kingdom. We were interested in understanding the personal experience of an intervention that was new and unfamiliar in this context. Narrative interviewing was identified as an appropriate method to address the aims and objectives of the study. We selected a core interview question to guide the narrative interviews. The question was as follows: ‘Tell me about your experiences of face masks since they were made mandatory on public transport and in healthcare settings on the 15 June 2020’. We then used unstructured prompting as necessary to provide further clarification or detail to that overall interview question. At the time of the study, face‐to‐face interviews were prohibited by law. We were conscious of the potential to omit the experiences of some groups—including, for example, those who are D/deaf or neurodiverse—if interviewing took place exclusively through telephone/video calls. We, therefore, offered a choice of email or telephone interviews to participants to maximise inclusivity. Interviewing was conducted by (K. F., P. C., A. C., G. M. and E. H.). All telephone interviews were transcribed verbatim to produce written transcripts, then analysed alongside the written email interview accounts. The National Institute for Health and Care Research Equality in research checklist informed the design of the study (https://mk0qebimerabt73npg13.kinstacdn.com/wp-content/uploads/2020/07/Checklist_COVID_BME_v2.pdf). An easy‐read participant information sheet, with visual cues and simplified text, was produced to complement the traditional information sheet for the study. The study received ethical approval from De Montfort University.

### 2.2 Participants

We sought a theoretically informed sample, based on the developing categories and emerging theory^15^ of our preliminary engagement in and monitoring of the developments in academic and public discourse around face masks and Covid‐19. We were particularly interested in including those with existing disabilities, hearing issues or learning differences, who identified as Black, Asian or minority ethnic, or who were on a low income. Recruitment for the study was primarily achieved through advertisement on social media (Twitter) and through relevant charities and third‐sector organisations which were active with the groups we hoped to include. These included charities supporting those who were D/deaf, such as Deaf Action, and organisations supporting those living with disabilities, such as Shaping our lives and Finding your Feet.

Participants were all aged 18 and over and lived in the United Kingdom. The final sample comprised 40 participants; 15 completed email interviews and 25 telephone interviews. Both forms of data generation elicited rich narrative accounts. As with verbal testimony, the length of email responses varied, but we used follow‐up questions to elicit additional information or clarification much as we would within the telephone interviews. Those who were D/deaf or had neurodiversity appeared more likely to request participation via email to make their participation possible and more accessible to them. We offered both options to all participants and did not ask why they had chosen one over the other, as we felt it was important for inclusivity to not make the choice of email conditional on a particular condition or disability. The data were generated between 29 July and 6 October 2020: early in the Covid‐19 pandemic, and soon after the introduction of face mask mandates in England and Scotland from June 2020, and in Wales and Northern Ireland from July (https://www.gov.uk/government/news/new-rules-on-face-coverings-coming-in-on-monday-will-help-keep-passengers-safe). Our work predated both the authorisation of the first Covid‐19 vaccine by the UK's Medicines and Healthcare Products Regulatory Agency in December 2020 and the spread of more infectious variants of the virus from winter 2020 onward. At the time of the interviews, the United Kingdom had experienced one ‘lockdown’ which then was followed by a partial reopening of some businesses and facilities during the summer months. A further lockdown then occurred in the autumn of 2020. Covid‐19 cases remained high during the period of data generation and knowledge of its transmission was still developing. A £20 high street shopping voucher to thank participants for their time was given on completion of participation.

Participants were asked to complete a short anonymous demographic survey on completion of their interview (via a weblink). Thirty‐nine of the 40 participants completed this (see Table 1). Twenty‐eight were female and 11 were male. Twenty‐nine people were identified as disabled, with 11 having a psychological or mental health condition. Almost half the sample was on household incomes of £20,000 per annum or less, with 23% on less than £10,000 pa. Seventy‐five percent of the sample identified as being white, with 16% from a range of minority ethnic backgrounds; 47% were of the Christian faith. Although we did not systematically collect data on their location, recruitment was nationwide, narrative data indicated that participants were from across the United Kingdom and from both rural and urban locations.

**Table 1:** Demographic characteristics of the sample.

Characteristic	*n* (%)
Male sex	11 (28)
Female sex	28 (72)
Gender identity matches the biological sex	39 (100)
Identifies as disabled	29 (74)
Longstanding psychological or mental health condition	11 (33)
Health issues impairing mobility	10 (30)
D/deaf or serious hearing impairment	4 (12)
Religion: Christian	18 (47)
Religion: Hindu	2 (5)
Religion: Jewish	2 (5)
Religion: Sikh	1 (3)
Religion: None	13 (33)
Religion: Other	2 (5)
Ethnicity: Asian Bangladeshi	1 (3)
Ethnicity: Black British	1 (3)
Ethnicity: Indian	4 (10)
Ethnicity: White British	26 (67)
Ethnicity: White other	3 (8)
Income: Less than £10,000 per annum	9 (23)
Income £10,001–£20,000 per annum	10 (26)
Income: £20,001–£30,000 per annum	5 (12)
Income: £30,001–£40,000 per annum	4 (10)
Income: £40,001–£50,000 per annum	2 (5)
Income: More than £50,000 per annum	7 (18)

### 2.3 Analysis

Data were analysed using reflexive thematic analysis following the process described by Braun and Clarke.^16^ The data were analysed for what was said as well as how it was said, using inductive coding and then the generation of broad themes. E. H. and G. M. conducted the initial coding and naming of themes before the agreement with the wider authorship team. For the purposes of this paper, we focus on three themes: the emotional aspects of face mask use; health and safety issues and the societal impacts of face masks.

## 3 FINDINGS

### 3.1 Emotional aspects of face mask use

Face masks were often reported as being entangled with other emotive aspects of Covid‐19. The fear and uncertainty of the pandemic were sometimes visible in narratives of the face mask. Some felt that face masks created new stresses or worries for them. For some, these stresses could translate into physical difficulties; for others, notably those with hearing impairments, they created social challenges:

When you have got a mask on, all the stress breathing in and breathing out, I think you realise it goes back internally so it gets you more stressed. (P13)

My experience of face coverings is obviously horrific because you are taking away the bits that I try and rely on for cues and information when I am with people. It's OKish if I know somebody well but if I don't know them it's a minefield for me. (P1)

Others found that communication improved as a result of face masks, as it meant that those around them had to use different means of communicating given that facial gestures were less apparent:

I communicate a lot using gestures because it's kind of all I have. The rest of the world aren't used to doing that and now they have to, so it's brought them out of their comfort zone into mine. And as a result I found mask wearing quite a positive thing. (P22)

Many participants felt more secure when wearing a face mask in public settings. Experiences during the early parts of the pandemic had created fear for some about the risks of the virus. Masks were seen as one means of restoring safety:

I felt quite anxious going out to public places at first but it definitely helps to reduce that and provides a sense of security. I prefer to see other people wearing them too, although understand that for some people it's not possible. The more I've been wearing it, the more comfortable I've started to find it. (P35 [email])

If you are wearing a mask then you do feel less worried about maintaining social distance I guess. Because you do feel as if you have got that covered in a way. So you know if I was out in a shop and someone brushed by me and we are both wearing masks then I don't think I would feel, I wouldn't feel as anxious or worried that that has, that closeness has compromised the situation. (P20)

Anxiety about the disease itself was therefore often closely entangled with emotions towards face masks. This included participants' attitudes towards others wearing (or not wearing) masks. For example:

I would never think twice about wearing it in a public space with strangers, I don't know them and it feels dangerous. (P18)

I am disappointed that others do not wear masks. I feel it is selfish. (P34 [email])

However, not all participants felt safer as a result of face masks. Moreover, they contributed to the social dynamics of the pandemic that, for some participants, could generate more challenge than comfort. For some, anxiety around their use, associated with their own difficulties in wearing them or with the risk of confrontation if they did not wear them, created challenges:

I notice my, you know, my breathing and that can have a knock‐on effect in terms of starting to feel quite anxious. And then when I notice I am becoming anxious I then develop a nervous cough and so in this kind of climate where you are worried about what people are thinking, when I start developing a nervous cough then I start to become more paranoid thinking people are looking at me, they are making judgements they are assuming that I might have Covid. (P20)

I get anxious and a lot of anxiety when I wear one, especially on the bus, I get panic attacks and scared, I am afraid to look at people and I think I am going to get arrested, I know it's [sic] sounds silly and I have see [sic] a lot of things like that happened to me a very long time and it bring back memories, my emotions are scared, angry and afraid of people. (P32 [email])

While those expressing such concerns were in the minority, the range of emotions and experiences that people reported around the use of masks was notable.

### 3.2 Health, safety and communication issues

Physical issues related to the use of face masks were often reported by participants. Some were seen as minor; others caused much greater issues or made for a more disabling context. Vision impacts were commonly reported; they often related to the ‘misting’ of glasses when wearing a mask:

I've got to wear a face mask every day. Which is alright but I wear glasses so the glasses steam up and it's a nuisance really but it's something that you've got to do. (P5)

The challenge of keeping glasses clear when wearing a mask sometimes meant that participants felt they had to move their masks away from their faces but often felt conscious about doing this for fear of what others may think:

I wear glasses as well and I have a cloth mask so every time I breath it all steams up so I end up, I am in a wheelchair as well so if I go out with my wife I am pulling it away from my nose a bit so it gets a bit more air around. Then you get strange looks because you are doing that. ‘If you are going to wear it then wear it’. That's the feeling you get sometimes. (P14)

The issue of glasses ‘fogging up’ was generally portrayed as an inconvenience by participants when they were moving around on foot or in a wheelchair or in retail settings, but some did worry about the impact of mask use on their peripheral vision and its consequences for their safety:

Sometimes when I am walking downstairs, I am accident prone, I haven't got much good balance because when I fell down and broke my arm, my other arm was fractured as well, I find it difficult walking straight, I lose my balance as well. So when walking down steps I just look down where I am putting my foot down and because of the mask sometimes I have to hold my mask away and then look down. (P12)

Most participants had solutions for issues with vision and saw the challenges presented by mask use as too minor to impact their activities overall. Similarly, participants reported skin irritation as a result of using face masks, but some at least were able to mitigate this:

My skin would break out around my chin area. And it did take a while for those spots to go out which was quite irritating. At one point they did get quite painful but I don't know how they have not made an appearance yet but I have got them under control at the moment. That's the only irritation I have experienced. I think that's quite common, acne caused by face masks. (P23)

I have a skin condition… I found that the surgical face masks the blue ones they irritated my chin and my skin more and made it itch basically. So, I could either go without one or I have got a fabric one now. So that's a lot more comfortable…it still causes some irritation but it's a lot less. (P3)

Other participants found that wearing a mask caused problems with their heat regulation; this in turn could exacerbate existing health issues:

They make me very hot, I know they make everybody very hot, but I am obese and also I have got various different health conditions that give me temperature regulation issues. (P2)

I have problems regulating my temperature and so like on a hot day I find it really hard to cool myself down. So, on a hot day having to wear a face mask I can get really really hot which then causes my diabetes or sugars to drop suddenly. So, I was having a lot of hypos in work simply from maybe I was [conducting a particular work task] and it was a hot day and I was stressed and I couldn't regulate my temperature. And then I was panicking and then my sugars were just phew and that's it and that's me off the floor then having to go and sort that out. (P19)

Other physical difficulties were more common, and feelings of difficulty with breathing and a sense of panic when wearing a face mask were reported by several participants. Some had pre‐existing respiratory issues and tried face masks before finding that they exacerbated their existing conditions:

I wore my mask but actually I was really out of breath and I felt really like I couldn't breathe with it on. And that disappointed me because I thought I would feel totally fine with it. And I had to take it off because I just felt like I couldn't breathe at all with it on. And those two really have, oh since then I've tried again to wear the mask in a shop and it was fine but I had to keep kind of lifting it up from my chin to create a bit of air. … I have chest problems which is why I was shielding in the first place. So, I think although I have masks I think I will probably wear them less than I thought. (P15)

The first experience was in June, when they were first made mandatory only on trains. I took a train journey of roughly 1 hour, and 10 minutes in I had to lower the mask to breathe. It was also a very hot day, and the train windows simply insulate the heat from the sun into the enclosed environment, much like car windows. I ended up having to create a behaviour pattern of lowering the mask, catching my breath, and putting it back just to stop myself from feeling faint. (P27 [email])

For some, the difficulties experienced with breathing when wearing a mask were severe enough to limit their activity outside their homes:

I'm distressed that I don't feel I have an option and so it's making my breathing [difficult], I have panic attacks, so it's made me have a panic attack in the shop. So, whenever I'm down an aisle I pull it away so that I can take a deep breath. Luckily I only go out for the odd one or two things in the shop but it makes that journey or that activity so horrific. (P4)

I then ordered online some different types of mask, things that roll up from your neck and cover your face, cyclists wear them I think. I had hoped this would be better as it's not tight round your face, but again I felt a sense of panic and my heart was pounding. I did a headspace meditation app for calm, tried the face masks a few times to see if I could get used to it and breath properly, tried Bach flower rescue remedy, but it's not any better I'm afraid. gasping for air as soon as I take it off. (P36 [email])

Communication issues were also consequential for participants. For some this was about day‐to‐day interactions lost as a result of the pandemic, coupled with the use of masks:

I think it's odd, I mean you can't say much, if you pass people that you know it's very difficult to chat to them, they don't stop to talk for very long now. Usually you meet someone on the street and you have had a really good chat. Well all that's gone, people are really anxious to get away. So that has changed, it's changed your life totally I think. And you do feel cut off but I suppose we will have to get used to it. (P24)

For those with minor hearing difficulties, the use of face masks could generate awkwardness in day‐to‐day interaction. Problems could be much more significant for D/deaf people, who sometimes found themselves unable to participate fully in communication with others when in public spaces:

I do wear them in shops. I prefer it when shop assistants wear clear visors as it's easier to understand them although I don't lipread too well I can usually understand what they mean. If they wear a mask that totally covers their face it can be a problem if they try and speak to me. (P28 [email])

Suddenly all my colleagues faces were covered with masks and I could no longer lip read. I suppose I had forgotten how dependent I am on using facial expressions and lip reading on a daily basis at work and suddenly that option had gone. (P29 [email])

The health, safety and communication issues related to mask use were thus varied, ranging from minor inconveniences to problems that caused significant problems in day‐to‐day life, or even discouraged participants from engaging in their usual activities.

### 3.3 Societal issues

A range of broader societal issues also arose in participants' narratives. A major theme was the impact of face masks on people's activities, including how often they engaged in social activities such as shopping, travelling or interacting with other people. This applied as much to those who did not routinely wear masks as to those who did. The world of some participants had contracted due to fear of being challenged in public settings about their exemption from wearing a mask:

So for me it's just every element of anxiety inducing. … For somebody who can't wear one it's essentially cut me off from, I have lost my hairdresser, all sorts of places that I have been to for years. And continuity is very important for me so to me it's like my world is closing in. (P1)

I'm now planning on doing online shopping only. I feel I should try to be brave and go again, but I don't feel ready to try it again unless I get desperate. For example, today I am going past the shops on my way home from work, and would like to pop in and get a few bits, but I daren't. Annoyed with myself for being a coward. My partner will make a separate 6 mile journey to go shopping which is inconvenient. (P36 [email])

Others found mask‐wearing difficult but persevered, sometimes making fewer trips out of the house to avoid having to wear a face mask:

Well if I am honest because I find it uncomfortable I don't go out as much as I would like to just because it is not as pleasant as it used to be. … And sometimes I choose to stay at home rather than feel you know, because the anxiety you have with the face mask, it can be very overwhelming. And so I just want to feel that way so I have to limit my amount of time that I spend outside. (P16)

Some participants directly related their decision to limit their trips out to the use of a mask, for example where it caused them specific physical issues or increased anxiety, as for participant 16 in the quote above and participant 4 below; for others decisions to limit excursions may have also been influenced by a general fear of Covid‐19. We did not directly ask for information about fear of Covid in the interviews we conducted; for some the general concerns they had due to the pandemic as a whole may have been conflated with fears relating to masks specifically. This lived experience remains an important part of understanding their perspectives on face mask use.

Some had modified their social practices to try to ease their discomfort or stress around mask‐wearing, carefully timing excursions outside the house or taking different, quieter routes to shops, for example:

It makes going out or popping into a shop to have a browse, it's not something I do any more. I call in specifically for an item you know, it's taken that enjoyment out of walking around a shop having a look and just seeing what's there… I have got it all timed now, you shouldn't have to do that, I should be able to go out anytime I feel shouldn't I. So yes, it's been very restrictive. (P4)

Face masks were introduced as part of a raft of measures against Covid‐19, exemplified in the UK government's slogan ‘Hands, Face, Space’. Some participants, however, suggested that face masks reduced people's propensity for social distancing, intensifying rather than alleviating their sense of risk:

But what I actually notice the second people have masks on, and this is professionals, this is people on telly, all sorts of people, they automatically go nearer you anyway because they have got the mask on; it gives a false sense of security. (P1)

Other people wearing masks sometimes present another problem. Some people seem to think that wearing the wearing [sic] of masks means social distancing is not required and they don't take care in the supermarket (the only shops I go in) to give one space. (P31 [email])

Face masks may then have additional implications for other social measures designed to limit the spread of Covid‐19. For some, this was a cause for concern, but others felt more relaxed about occasional close proximity to others when faces were covered.

The cost of face masks was also a concern for some participants. As noted above, 23% of the sample were on incomes under £10,000 per annum, and many were on benefits. Consequently, they had limited means to purchase face masks. Some felt that cost should have been given greater consideration with the introduction of the policy:

We shouldn't have to pay for them either: they should be distributed at certain places where you can just pick them up. I'm someone on benefits: I don't have any extra money to buy, no I think it's really wrong. (P4)

I think I paid something like £6 per mask when I got the first batch and I got a couple for myself and a couple for family members. So, I am sure if you are on benefits £6 might be a lot but I think there is a case maybe for the government handing them out to people who are receiving Universal Credit or on free school meals, I don't see why you wouldn't do that. (P8)

Laundering reusable face masks added to the challenges and costs identified by participants. As one noted, the use of cloth (reusable) face masks ‘generates a bit of extra washing’ (P2). Some had found ways to manage this, but for others, it remained a concern, particularly the need to wash masks at a high temperature and the cost of doing so:

The material ones, they say you have got to wash them at 60 degrees. … I live on my own so I am not going to have a 60 degree wash that much and it costs a lot for a washing machine. People forget those things. (P13)

Additional washing may also have environmental impacts, through increased water and energy usage. Participants discussed further environmental concerns around face mask use, often centred on disposable face coverings and their ubiquitous appearance as litter:

I was worried about the amount of waste involved and sure enough we now see them dropped on the street all over the place. (P8)

I now get cross from an environmental point of view, you see them discarded in random places like in the woods, we went for a jog and I was like ‘What, who you are distancing from in the woods’– it's so weird. (P18)

While more recent advice has cast doubt on the effectiveness of cloth‐based masks,^17^ concerns about the environment had led some to choose reusable face masks in preference to disposable ones:

[The pharmacy] had the boxes of the disposable ones and I thought shall I buy some. And I thought no because it's just, I don't agree with, well I try to minimise how much disposable stuff I have, I have a keep cut and stuff like that. And the idea of buying a load of masks and then just chucking them away is not very environmentally sound. (P17)

## 4 DISCUSSION

Our study demonstrates a wide range of issues and experiences relating to the use of face masks during the Covid‐19 pandemic. Not all were negative. Some participants found that masks made them feel safer during a period of uncertainty. Many also reported continuing to wear face masks, even when facing distress or other issues as a result of using them. The positive benefits of stopping the spread of disease and wanting to be a ‘good citizen’ were largely identified as the drivers of such perseverance. Nevertheless, our participants highlighted emotional, physical and social issues relating to the use of masks in their narratives. The focus of the interviews on individual experiences, and the approach to sampling and recruitment, may have meant that these issues were more prominent in our data than a discussion of the potential value of masks in slowing the spread of Covid‐19. Some issues were specific to those living with particular health conditions, but others were more widespread, for example, difficulties with glasses or with finding affordable masks. Our data show how face masks may create direct and indirect challenges for individuals, but also highlight how people have persevered with the use of masks out of a desire for safety for themselves, and others, from Covid‐19. We have not explored the possible potential political or ideological dimensions of the reasonings within this paper but we have written about this elsewhere.^18^

A notable feature of our data set was the binary characteristic of some of the emotions expressed around mask use. For example, while for some participants (such as those with past experiences of trauma) masked faces provoked fear, others reported an increased sense of safety as a result of masks being introduced. Likewise, many reported debilitating anxiety from using a face mask, yet others found security in wearing a mask. Emotional messaging is often used in public health campaigns and can be key in influencing changes in behaviour,^18^ but our findings suggest that such efforts may provoke a divergent range of responses across different sections of the public.

Face masks as an intervention against Covid‐19 have been the topic of much scientific and public debate during the pandemic. While concerns were raised by some about the potential unintended consequences of face masks,^11^ others have argued that their potential advantages likely outweighed any negative consequences.^19^ Our findings demonstrate myriad unintended consequences associated with the use of masks, including areas of concern as varied as physical and mental health, affordability, accessibility, access to washing facilities as well as environmental impacts. Undoubtedly, some of these issues will be of trivial importance to many; our approach to sampling and recruitment and the nature of our data set mean that we cannot comment on the population‐level impact of these problems. For some individuals, however, their consequences are far from insignificant, and their impact could extend into important aspects of their day‐to‐day lives. Moreover, some of those vulnerable to these impacts were already in marginalised groups, such as those with mental health disorders and those on low incomes. Several participants expressed concern about the affordability of masks, reflecting the large proportion of our sample on low incomes. As Frohlich and Potvin note, ensuring that efforts to improve public health do not exacerbate social exclusion is vital:

That the objective of improving population health may not necessarily be compatible with the objective of reducing health disparities is becoming acknowledged in an increasing number of health policies. One way to ensure that vulnerable populations are not left behind in the improvement of population health is to distinguish these objectives and design public health strategies that use both population and vulnerable population approaches to interventions^20^

Adaptations to the rollout of masks, including making them free at the point of use in settings where required, as embraced for example by authorities in France and Italy,^21^ might help to mitigate such impacts.

However, not all negative impacts will be so easily neutralised and cataloguing the potential and actual unintended consequences of public health interventions is vital. The empirical examination helps to reveal issues—such as risks of social exclusion caused by masks directly, or by withdrawal from day‐to‐day social activities—that may otherwise remain hidden. Mapping our findings against Lorenc and Oliver's framework of adverse effects of public health interventions^22^—which covers direct harms, psychological harms, equity harms, group and social harms and opportunity cost harms—shows that the impacts of mask use fall into several of these domains. For example, affordability concerns may represent a form of equity harm, while direct harms included skin complaints, breathing issues, communication challenges and mobility difficulties. Psychological harms were discussed extensively by participants, including anxiety relating to one's own mask usage, and both use and nonuse by others. As Lorenc and Oliver note

Whether intended or unintended, direct or indirect, interventions of any kind are likely to have wider effects than usually acknowledged by evaluators. For ethical and methodological reasons, it is imperative that the harmful effects of interventions are considered, collected and if possible alleviated by evaluators and designers of interventions^22^

This paper thus begins to identify the unintended consequences of the use of face masks during the Covid‐19 pandemic, particularly as they are experienced by marginalised groups. Where possible, the unintended consequences of all public health interventions should be considered and addressed before interventions are implemented; where the urgency of the situation precludes this, it is incumbent on researchers and policymakers to investigate them promptly and thoroughly, to inform speedy efforts to mitigate and manage any harm.

Our study has some limitations. While our qualitative approach is helpful in identifying the range of issues that face masks and mask mandates may present, particularly for marginalised groups, it does not offer insight into the prevalence of these problems. Participants were a self‐selecting sample who may have had a particular interest in sharing their views on masks. Our narrative approach sought to ensure that interviews were led by participants rather than solely by researcher interests. The study is bound by the geographical context of our participants, and research in other countries would likely have different relationships with face masks given divergent prevailing attitudes towards mask use.^7^

## 5 CONCLUSION

Our study provides much‐needed evidence about perceptions of and experiences of face masks in the United Kingdom and characterises some of the harms that masks may cause, particularly for groups who may be at risk of marginalisation through mask mandates. Our findings demonstrate the value of in‐depth, qualitative insights from a diverse population in understanding public health interventions, and offer insights that may be important in making rational, equitable decisions about the use of masks and mandates, including weighing benefits and harms, ensuring that the downsides of masking interventions are appropriately mitigated, and ensuring that public health policy accounts for the needs of marginalised groups.

## AUTHOR CONTRIBUTIONS

Esmée Hanna designed and delivered the study, including its administration and management, and completed the analysis and writing of the original draft. Graham Martin designed the original study and completed the analysis and writing of the original draft. Anne Campbell conducted fieldwork for the investigation and contributed to the writing (editing and reviewing). Paris Connolly conducted fieldwork for the investigation and contributed to the writing (editing and reviewing). Kristine Fearon conducted fieldwork for the investigation and contributed to the writing (editing and reviewing).

## CONFLICT OF INTEREST STATEMENT

The authors declare no conflict of interest.

## ETHICS STATEMENT

The study received ethical approval from De Montfort University, Leicester.

## Data Availability

Due to the nature of the research, supporting data are not available for ethical reasons. The participants of this study did not give written consent for their data to be shared publicly.

## References

[1] Cheng KK , Lam TH , Leung CC . Wearing face masks in the community during the COVID‐19 pandemic: altruism and solidarity. Lancet. 2022;399:e39‐e40. 10.1016/S0140-6736(20)30918-1 32305074 PMC7162638

[2] Howard J , Huang A , Li Z , et al. An evidence review of face masks against COVID‐19. Proc Natl Acad of Sci. 2021;118(4):e2014564118. 33431650 10.1073/pnas.2014564118 PMC7848583

[3] Carbon CC . Wearing face masks strongly confuses counterparts in reading emotions. Front Psychol. 2020;11:566886. 33101135 10.3389/fpsyg.2020.566886 PMC7545827

[4] World Health Organization (WHO) . Mask Use in the Context of COVID‐19: Interim Guidance. WHO; 2020.

[5] Dorfman D , Raz M . Mask exemptions during the COVID‐19 pandemic—a new frontier for clinicians. JAMA Health Forum. 2020;1(7):e200810. 10.1001/jamahealthforum.2020.0810 36218689

[6] Harte L . Plans to tighten rules on wearing face coverings delayed by Stormont ministers. January 6, 2022. Accessed January 21, 2022. https://www.belfastlive.co.uk/news/northern-ireland/covid-ni-plans-tighten-rules-22668044

[7] Feng S , Shen C , Xia N , Song W , Fan M , Cowling BJ . Rational use of face masks in the COVID‐19 pandemic. Lancet Respir Med. 2020;8(5):434‐436. 10.1016/S2213-2600(20)30134-X 32203710 PMC7118603

[8] Hanna ES , Dingwall R , McCartney M , et al. Sociocultural reflections on face coverings must not ignore the negative consequences. BMJ. 2020;371:m3782. 10.1136/bmj.m3782 33004352

[9] Ramaci T , Barattucci M , Ledda C , Rapisarda V . Social stigma during COVID‐19 and its impact on HCWs outcomes. Sustainability. 2020;12(9):3834. 10.3390/su12093834

[10] Howard MC . Gender, face mask perceptions, and face mask wearing: are men being dangerous during the COVID‐19 pandemic? Pers Individ Dif. 2021;170:110417. 10.1016/j.paid.2020.110417 33052155 PMC7543707

[11] Martin GP , Hanna E , McCartney M , Dingwall R . Science, society, and policy in the face of uncertainty: reflections on the debate around face coverings for the public during COVID‐19. Crit Public Health. 2020;30(5):501‐508. 10.1080/09581596.2020.1797997

[12] Kohek J , Seth A , Edwards M , Zwicker J . Mandatory Mask Bylaws: Considerations beyond Exemption for Persons With Disabilities. Social Science Research Network. https://papers.ssrn.com/abstract=3696049

[13] Bakhit M , Krzyzaniak N , Scott AM , Clark J , Glasziou P , Del Mar C . Downsides of face masks and possible mitigation strategies: a systematic review and meta‐analysis. BMJ Open. 2021;11(2):e044364. 10.1136/bmjopen-2020-044364 PMC7903088 33619199

[14] O'Byrne L , Gavin B , McNicholas F . Medical students and COVID‐19: the need for pandemic preparedness. J Med Ethics. 2020;46(9):623‐626. 10.1136/medethics-2020-106353 32493713 PMC7316103

[15] Coyne I . Sampling in qualitative research. Purposeful and theoretical sampling; merging or clear boundaries? J Adv Nurs. 1997;26(3):623‐630. 10.1046/j.1365-2648.1997.t01-25-00999.x 9378886

[16] Braun V , Clarke V . Using thematic analysis in psychology. Qual Res Psychol. 2006;3(2):77‐101. 10.1191/1478088706qp063oa

[17] Abaluck J , Kwong LH , Styczynski A , et al. Impact of community masking on COVID‐19: a cluster‐randomized trial in Bangladesh. Science. 2022;375:eabi9069. 10.1126/science.abi9069 34855513 PMC9036942

[18] Hanna E , Martin G , Campbell A , Connolly P , Fearon K , Markham S . Experiences of face mask use during the COVID‐19 pandemic: a qualitative study. Sociol Health Illn. 2022;44(9):1481‐1499. 10.1111/1467-9566.13525 36040759 PMC9538649

[19] Heffner J , Vives ML , FeldmanHall O . Emotional responses to prosocial messages increase willingness to self‐isolate during the COVID‐19 pandemic. Pers Individ Dif. 2021;170:110420. 10.1016/j.paid.2020.110420 33082614 PMC7561320

[20] Greenhalgh T , Schmid MB , Czypionka T , Bassler D , Gruer L . Face masks for the public during the Covid‐19 crisis. BMJ. 2020;369:m1435. 10.1136/bmj.m1435 32273267

[21] Frohlich KL , Potvin L . Transcending the known in public health practice. Am J Public Health. 2008;98(2):216‐221. 10.2105/AJPH.2007.114777 18172133 PMC2376882

[22] Lorenc T , Oliver K . Adverse effects of public health interventions: a conceptual framework. J Epidemiol Community Health. 2014;68(3):288‐290. 10.1136/jech-2013-203118 24336237

